# Sensing nanoscale electromagnetic forces when the heat is on

**DOI:** 10.1093/nsr/nwae184

**Published:** 2024-05-27

**Authors:** Eric O Potma

**Affiliations:** Department of Chemistry, University of California, Irvine, CA 92697, USA

In scanning probe microscopy (SPM), a sharp tip scans across a sample to produce an image with nanometer-scale resolution or better. SPM contrast is derived from the physical interplay between the tip and the sample. A special class of SPM techniques measures forces when the tip–sample junction is illuminated by an external light source. Photo-induced forces can be sensitive to the optical properties of the sample, thus enabling nanoscale imaging with spectroscopic, material-specific contrast.

The family of light-based SPM techniques has grown over the years and various applications have emerged. One application focuses on thin materials using mid-infrared (MIR) light to record images based on the vibrational or phonon-like modes of the sample [1]. Several processes can contribute to the measured force signal and chief among these is the photo-thermal (PT) expansion force. This effect follows from the temperature increase due to the heat deposited into the material after MIR absorption.

The prominence of PT expansion forces under MIR illumination has somewhat overshadowed the contribution of other light-induced forces. Nonetheless, there are scenarios in which the PT force is rather weak and other mechanisms may come to dictate the measured force signal. A prime example is the electromagnetic force that results from the light-induced motions of charges in the tip and the sample, which, in the dipole approximation, is referred to as the photo-induced dipole force (PiDF). The latter force has properties that are different from those of the PT force, such as its sharper dependence on the tip–sample distance and its relative insensitivity to sample thickness. In the visible range of the spectrum, the PiDF can be the dominant light-induced force when visualizing nanoscale structures that exhibit strong electronic polarizabilities [2]. In the MIR range, however, the PiDF is considered weak and its role in nanoscale imaging negligible [3].

Yet, in a recent paper, Li *et al.* show that, under certain conditions, it is the PiDF that prevails [4]. They observe that the Si–O–Si lattice vibrations of a quartz substrate can couple efficiently to the MIR field confined by the tip, forming a light–matter resonance mode known as a surface phonon polariton. This strong resonance condition, in turn, gives rise to a PiDF that trumps the measured PT expansion force by an order of magnitude. While this condition may appear exotic, the authors point out that quartz is a generic substrate material and that the proposed excitation scheme can be leveraged for a wide range of nanoscale imaging applications. For instance, they demonstrate that the strong quartz response can be modulated by layering thin materials on top of the substrate, giving rise to a reduced signal due to the sharp distance dependence of the measured PiDF. The resulting variation in signal provides a high-resolution morphological map of the layered material. These are precisely the conditions under which the PT expansion force struggles, as the physical expansion of nanometer-thick materials approaches the detection limit of the instrument.

The work by Li *et al.* underlines that electromagnetic forces in MIR-based force microscopy can produce meaningful signals, even in the presence of light-induced sample heating.
